# Leukocytoclastic vasculitis after exposure to COVID-19 vaccine^☆^

**DOI:** 10.1016/j.abd.2021.09.003

**Published:** 2021-11-10

**Authors:** Matheus Fritzen, Gabriella Di Giunta Funchal, Mariana Oliveira Luiz, Giovanna Steffenello Durigon

**Affiliations:** a Faculty of Medicine, Universidade Federal de Santa Catarina, Florianópolis, SC, Brazil; b Department of Dermatopathology, Universidade Federal de Santa Catarina, Florianópolis, SC, Brazil; c Department of Dermatology, Universidade Federal de Santa Catarina, Florianópolis, SC, Brazil; d Department of Hematology, Universidade Federal de Santa Catarina, Florianópolis, SC, Brazil

Dear Editor,

A 60-year-old female patient with a history of chronic liver disease, portal hypertension, polycythemia vera, hypothyroidism, and type 2 diabetes mellitus, presented to the Emergency Section of a University Hospital reporting the appearance of painful purpuric lesions in the lower limbs three days before. She denied fever, chills, arthralgia, or trauma. She denied the use of new medications. She reported receiving the second dose of the COVID-19 vaccine (Oxford-AstraZeneca) approximately eleven days before. She denied a similar previous clinical picture. On physical examination, she had purpuric lesions and palpable papules, which did not disappear on digital pressure (Figure 1, Figure 2).

**Figure 1:**
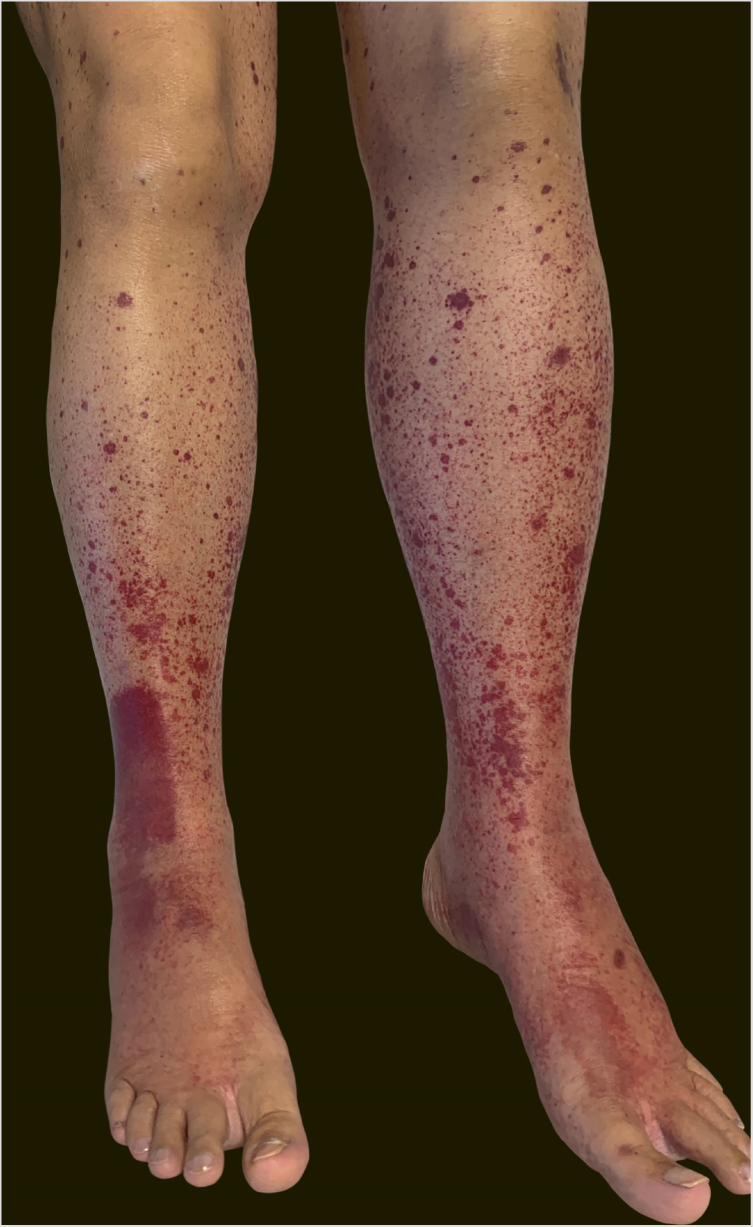
Purpuric lesions on the lower limbs on the first day of hospitalization.

**Figure 2:**
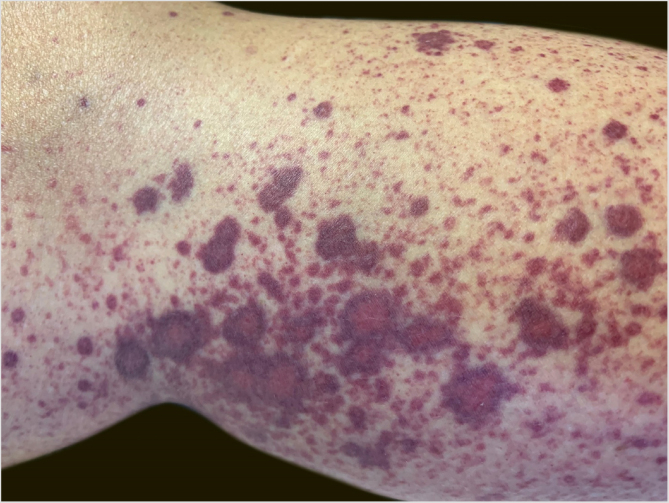
Multiple petechiae and palpable purpura.

She reported daily use of propranolol, metformin and levothyroxine. She described stability of the polycythemia vera since December 2015. She was submitted to two lower-limb skin punch biopsies for histopathological analysis and immunofluorescence (IF). Prednisone (1 mg/kg/day) was started once a day. The histopathological examination showed a mixed inflammatory infiltrate with predominantly perivascular fragmented neutrophils associated with extravasated red blood cells (Fig. 3a–c). IF showed deposits of IgA and IgM on the walls of postcapillary vessels (Fig. 3d). The histological picture was compatible with leukocytoclastic vasculitis. The patient denied symptoms or a previous clinical picture of COVID-19. The possibility of cryoglobulinemia was suggested, and serum cryoglobulins were measured, which were negative. She had elevated C-reactive protein levels and leukocytosis with a leftward shift. The remaining blood count results, liver function, coagulogram, and partial urine tests were within the normal limits or compatible with the comorbidities (Table 1). After three days of hospitalization, she showed improvement of the lower limb lesions and painful symptoms. After a seven-day treatment with 60 mg prednisone, a progressive reduction was started, and she was discharged from the hospital on 40 mg/day of prednisone.

**Figure 3:**
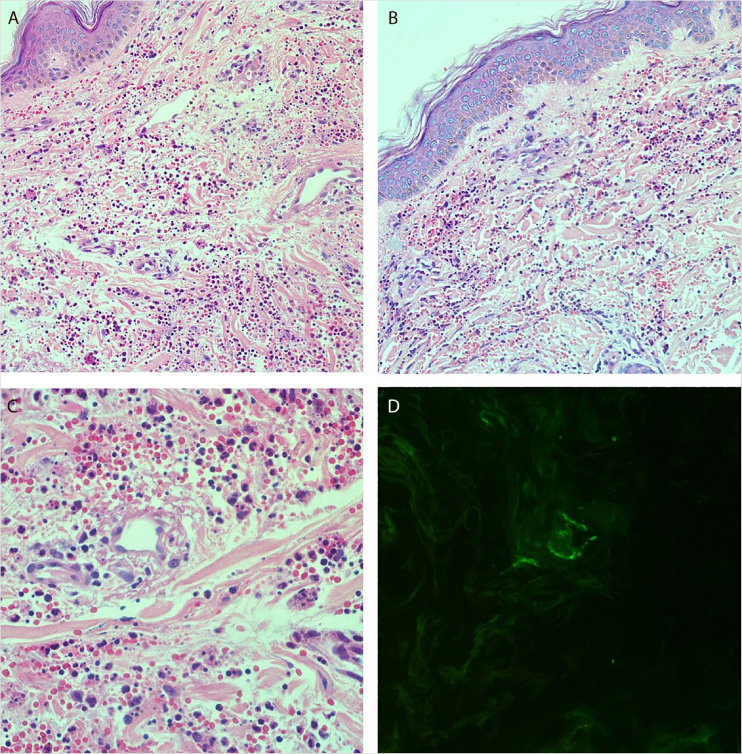
(A and B), Histopathology of the skin showing diffuse inflammatory infiltrate in the dermis (Hematoxylin & eosin, ×100). (C), Postcapillary dermal vessels permeated by neutrophils with leukocytoclasia. Presence of signs of endothelial damage with mural and interstitial fibrin deposits, in addition to extravasated red blood cells. (Hematoxylin & eosin, ×400). (D), Direct immunofluorescence showing granular deposits of IgA and IgM on the postcapillary vessel wall.

**Table 1:** Laboratory tests.^a^

Variables	Reference values (adults)	Admission
**Blood**		
Hematocrit (%)	11.5–16.5	13.6
Hemoglobin (g/dL)	36–48	43.2
Leukocytes (per μL)	3,000–11,000	31,610
Differential (mm^3^)		
Neutrophils	1,700–7,500	19,914
Lymphocytes	1,000–2,500	1,896
Monocytes	200–920	632
Eosinophils	20–670	632
Band cells	–	4,741
Metamyelocytes	–	2,528
Myelocytes	–	948
Promyelocytes	–	316
Platelets (per μL)	150,000–440,000	138,000
Prothrombin time (s)	12.1	16.8
INR	<1.2	1.42
Activated partial prothrombin time (s)	27.3	41.3
C-Reactive Protein (mg/L)	< 3	4.8
Erythrocyte sedimentation rate (mm/h)	Up to 15	1
Aspartate aminotransferase (U/L)	10–40	51
Alanine aminotransferase (U/L)	14–59	12
Lactic dehydrogenase (U/L)	81–234	1,304
Creatinine (mg/dL)	0.7–1.2	1.04

**Urine**		
Color	Citrine yellow	Citrine yellow
Aspect	Slightly cloudy	Slightly cloudy
Density	1,010–1,030	1,025
pH	5.0–7.0	5.0
Proteins	Negative	Negative
Reducing substances	Negative	Negative
Ketone bodies	Negative	Negative
Hemoglobin	Negative	Negative
Bilirubin	Negative	Negative
Urobilinogen	Negative	Negative
Epithelial cells	Rare	Rare
Leukocytes	<20,000/mL	56,000
Red blood cells	<20,000/mL	17,000
Bacterial flora	Absent	Discrete
Hyaline cylinders	<400/mL	2,000

a Reference values are affected by many variables, including the patient population and the laboratory methods used. The ranges used are for adult females who are not pregnant and have no medical conditions that could affect results. Therefore, they may not be adequate for all patients.

Immunization is a highly important resource in the fight against pandemics, especially the current one caused by COVID-19. However, possible side effects have not yet been fully described. As portrayed by Cohen et al. (2021) and Bostan et al. (2021), there are reports in the literature that point to leukocytoclastic vasculitis following the application of the COVID-19 messenger ribonucleic acid (mRNA) vaccine, such as the vaccine produced by Moderna and Pfizer.^1^, ^2^ However, there are few reports in the literature on these findings in cases of viral vector vaccines, such as the one produced by Oxford-AstraZeneca. Due to the patient’s multiple comorbidities, the investigation started with the main hypothesis of exacerbation of some of the previous diseases. Hence, the importance of considering possible reactions to the vaccine in the differential diagnosis.

It is unclear whether the COVID-19 vaccine can reactivate or trigger autoimmune diseases.^1^, ^2^, ^3^, ^4^ Leukocytoclastic vasculitis has been described in a range of infections and after administration of some vaccines, such as pneumococcal, influenza, rotavirus, hepatitis A and B, HPV.^3^, ^4^ We already know that SARS-CoV-2 can cause immune system hyperactivation through cross-reactivity and molecular mimicry,^5^ and it is possible that after the administration of the second dose of the vaccine, immune complexes comprising vaccine antigens and native antibodies initiated the vasculitis.^4^ The temporal association between the COVID-19 vaccine and the development of the skin condition is significant. This report suggests the possibility that the COVID-19 vaccine has the potential to induce leukocytoclastic vasculitis.

## Financial support

None declared.

## Authors' contributions

Matheus Fritzen: Statistical analysis; approval of the final version of the manuscript; design and planning of the study; drafting and editing of the manuscript; collection, analysis, and interpretation of data; effective participation in research orientation; intellectual participation in the propaedeutic and/or therapeutic conduct of the studied cases; critical review of the literature; critical review of the manuscript.

Gabriella Di Giunta Funchal: Statistical analysis; approval of the final version of the manuscript; design and planning of the study; drafting and editing of the manuscript; collection, analysis, and interpretation of data; effective participation in research orientation; intellectual participation in the propaedeutic and/or therapeutic conduct of the studied cases; critical review of the literature; critical review of the manuscript.

Mariana Oliveira Luiz: Statistical analysis; approval of the final version of the manuscript; design and planning of the study; drafting and editing of the manuscript; collection, analysis, and interpretation of data; effective participation in research orientation; intellectual participation in the propaedeutic and/or therapeutic conduct of the studied cases; critical review of the literature; critical review of the manuscript.

Giovanna Steffenello Durigon: Statistical analysis; approval of the final version of the manuscript; design and planning of the study; drafting and editing of the manuscript; collection, analysis, and interpretation of data; effective participation in research orientation; intellectual participation in the propaedeutic and/or therapeutic conduct of the studied cases; critical review of the literature; critical review of the manuscript.

## Conflicts of interest

None declared.
